# What the papers say

**DOI:** 10.1093/jhps/hnz033

**Published:** 2019-07-20

**Authors:** Ajay Malviya

**Affiliations:** Consultant Orthopaedic Surgeon, Northumbria Healthcare NHS Foundation Trust, Senior Lecturer, Regenerative Medicine—ICM, Newcastle University, 10 East Brunton Wynd, Newcastle upon Tyne, UK

The *Journal of Hip Preservation Surgery* (*JHPS*) is not the only place where work in the field of hip preservation may be published. Although our aim is to offer the best of the best, we continue to be fascinated by work that finds its way into journals other than our own. There is much to learn from it so *JHPS* has selected six recent and topical subjects for those who seek a summary of what is taking place in our ever-fascinating world of hip preservation. What you see here are the mildly edited abstracts of the original articles, to give them what *JHPS* hopes is a more readable feel. If you are pushed for time, what follows should take you no more than 10 min to read. So here goes …

## ANALYSIS OF THE REFERRAL PATTERN AND WAIT TIME FOR HIP ARTHROSCOPY IN A SINGLE-PAYER PUBLICLY FUNDED HEALTH CARE SYSTEM

Canadian researchers [1] set out to analyze the referral pattern for hip pain and to investigate the wait time for an orthopedic assessment by a hip arthroscopy surgeon in a single payer health care system. They hypothesized that a significant delay from time of onset of symptoms to time of assessment by a hip arthroscopy surgeon exists. This was a retrospective review of prospectively collected data in an academic hospital in a single payer health care system. An electronic database analysis was conducted searching for all referrals for hip pain between February 2017 and June 2017. Data were then analyzed with the aim to identify the most common reason for hip referral, calculate the duration of symptoms between onset and orthopedic assessment, and categorize previous investigations and treatments.

A total of 96 patients were included (47 male and 49 female). Main source of referrals was Family Medicine Physicians in 37% of cases and Primary Care Sports Medicine Physicians in 35%. The most common reason for referral was labral tear in 44.7% of cases followed by combined femoroacetabular impingement and labral tear in 21.8%. The duration of symptoms was longer than 2 years in 42% of cases and between 1 and 2 years in 40% of cases. Twenty percent of patients had previous intra-articular injection while 53% of patients had physiotherapy treatment (64% of patient underwent physiotherapy for longer than 6 months).

The authors concluded that in the Canadian single payer health care system, a significant delay from the time of onset of symptoms to the time of assessment by a hip arthroscopy surgeon exists with the vast majority of patients waiting more than 1 year. It is unknown if this delay affects the patient outcomes. They recommend a better screening process, centralized referrals to hip arthroscopy specialists, and appropriate patient work-up.

## WHAT IS THE MINIMAL CLINICALLY IMPORTANT DIFFERENCE AND SUBSTANTIAL CLINICAL BENEFIT VALUES FOR THE 12-ITEM INTERNATIONAL HIP OUTCOME TOOL?

In this multi-centered study, Martin *et al*. [2] have attempted to define minimal clinically important difference (MCID) and substantial clinical benefit (SCB) values for the 12-item International Hip Outcome Tool (iHOT-12) in patients undergoing hip arthroscopy for intra-articular pathology.

This was a retrospective review of prospectively collected data on patients who underwent hip arthroscopy. On initial assessment and follow-up between 335 and 395 days after surgery, subjects completed the iHOT-12 and a categorical self-rating of function (severely abnormal, abnormal, nearly normal or normal). One-half the standard deviation (SD) of the change in one-year iHOT-12 scores was used to calculate the MCID. Receiver operator characteristic analysis was performed to determine SCB values. A change in SCB value was determined based on an improvement in the categorical rating of function. Absolute postoperative SCB scores were calculated to determine scores that would be associated with normal function ratings or with abnormal or severely abnormal function ratings.

Of 1034 eligible patients, 733 (71%) met the inclusion criteria. The subjects consisted of 537 female patients (73%) and 196 male patients (27%), with a mean age of 35.3 years (SD 13 years). At a mean of 352 days (SD 21 days) after surgery, 536 patients (73%) were in the ‘improved’ group and 197 (27%) were in the ‘not improved’ group. The MCID was 13 points. An SCB change score of 28 points was able to identify patients who improved with high sensitivity (0.79) and specificity (0.72). Scores of 86 points or greater and 56 points or less were the cutoff values found to identify subjects who rated their function as normal and abnormal, respectively, with high sensitivity (0.74 and 0.90, respectively) and specificity (0.82 and 0.86, respectively).

This study has provided information to help interpret iHOT-12 scores for an ∼1-year follow-up period with MCID and SCB values of 13 and 28 points, respectively. In addition, a patient who scored 86 points or better was likely to have a normal rating of function, whereas a patient with a score of 56 points or less was likely to have an abnormal rating of function.

## IS THERE AN IMPROVEMENT IN SPORTS AND PHYSICAL ACTIVITY AFTER HIP ARTHROSCOPIC SURGERY FOR FEMOROACETABULAR IMPINGEMENT USING OBJECTIVE MEASURES?

Danish researchers [3] suggested that measurements of the physical activity level before and after hip arthroscopic surgery in patients with FAIS using both self-reported and objective accelerometer-based measures are lacking. They felt that comparing patients with a reference group of persons reporting no hip problems and conducting subgroup analyses investigating changes in physical activity level and self-reported outcomes according to pre-surgery activity level may further highlight the activity pattern for patients.

Sixty patients with FAIS eligible for hip arthroscopic surgery were consecutively included in a prospective cohort study (HAFAI cohort) together with 30 reference persons reporting no hip problems. Participants completed the Copenhagen Hip and Groin Outcome Score (HAGOS) together with questions regarding their sports activities. Furthermore, participants wore a three-axial accelerometer for five consecutive days during waking hours. The accelerometer-based data were analyzed and presented as total activity and type, frequency and duration of activities.

Patients experienced significant and clinically relevant changes in all HAGOS scores. An 88% of patients participated in some kind of sports activity 1 year after surgery. Overall, objectively measured physical activity did not change from before to 1 year after surgery. However, subgroup analyses of the most sedentary patients preoperatively revealed significant changes towards a more active pattern. Compared with reference persons, patients performed less bicycling and running.

Despite clinically relevant changes in self-reported outcomes, patients did not increase their overall physical activity level 1 year after surgery. Physical activity levels were lower in patients than in the reference group and patients continued bicycling and running less compared with the reference group.

## MILD OR BORDERLINE HIP DYSPLASIA: ARE WE CHARACTERIZING HIPS WITH A LATERAL CENTER-EDGE ANGLE BETWEEN 18° AND 25° APPROPRIATELY?

Controversy surrounds the classification and treatment of hips with a lateral center-edge angle (LCEA) between 18**°** and 25**°**. It remains undetermined as to whether periacetabular osteotomy (PAO) or arthroscopic surgery is best used to treat this patient population. Patients with hip pain and mild or borderline acetabular dysplasia defined by an LCEA between 18**°** and 25**°** have different features of acetabular and femoral morphology, as determined by other relevant radiographic measures assessing the anterior and posterior acetabular walls, anterior coverage of the femoral head by the acetabulum, and femoral head and neck junction sphericity.

In this retrospective review from Boston Children’s Hospital [4] patients who had an LCEA between 18**°** and 25**°** undergoing hip preservation between January 2010 and December 2015 with either PAO or hip arthroscopic surgery was performed. Anteroposterior, Dunn lateral, and false profile radiographs were used to measure the LCEA, Tönnis angle, anterior center-edge angle (ACEA), anterior wall index (AWI) and posterior wall index (PWI), femoral epiphyseal acetabular roof (FEAR) index and posterior to AWI, and alpha angle and femoral neck-shaft angle. An agglomerative hierarchical clustering analysis was then performed on the continuous radiographic variables to identify different subtypes of hip pathomorphology among the study cohort. There were sex-specific trends in hip morphology. Therefore, the authors proceeded to perform separate cluster analyses for each sex. Multivariate logistic regression was used to identify radiographic parameters for distinguishing between female patients who underwent hip arthroscopic surgery versus PAO.

Ninety-eight patients with hip pain and an LCEA between 18**°** and 25**°** underwent surgery in the study period, 77 (78%) were female, and 81 (82%) had complete radiographs for cluster analyses. The mean age was 22.6 years. Hip arthroscopic surgery was performed in 40 (41%) patients, and PAO was performed in 58 (59%) patients. The ACEA (45%), FEAR index (34%) and AWI (30%) were the most commonly abnormal radiographic parameters among all patients. In female patients, the ACEA (55%), FEAR index (42%) and AWI (34%) were the most commonly abnormal radiographic parameters. In male patients, the PWI (48%) was the most common radiographic abnormality. For female patients, three clusters representing different patterns of hip morphology were identified: acetabular deficiency with cam morphology, lateral acetabular deficiency, and anterolateral acetabular deficiency. For male patients, three clusters were also identified: posterolateral acetabular deficiency with global cam morphology, posterolateral acetabular deficiency with focal cam morphology, and lateral acetabular deficiency without cam morphology. The ACEA [odds ratio (OR) 47.7; *P* <0.001] and AWI (OR 3.9; *P* =0.049) were identified as independent factors predicting which procedure was performed in female patients.

The authors concluded that a comprehensive evaluation of radiographic parameters in patients with an LCEA between 18**°** and 25**°** identified sex-specific trends in hip morphology and showed a large proportion of dysplastic features among these patients. An isolated assessment of the LCEA is an oversimplistic approach that may jeopardize appropriate classification and may provide insufficient data to guide the treatment of hips with additional features of dysplasia and instability.

## NATURAL HISTORY OF THE DYSPLASTIC HIP FOLLOWING MODERN PAO

The purpose of this multi-center cohort study [5] was to delineate the long-term radiographic natural history of the dysplastic hip following PAO. The authors evaluated all patients undergoing PAO from 1996 to 2012, under expert surgeons based at three academic institutions in the United States. Inclusion criteria were PAO for DDH with a minimum 5-year radiographic follow-up. Exclusion criteria were PAO for isolated acetabular retroversion, neurogenic dysplasia, Legg-Calvé-Perthes disease, and prior hip surgery including osteotomies and arthroscopy. There were 288 patients, 83% of whom were women; the mean age and body mass index were 29 years and 25 kg m^−^^2^, respectively. The mean clinical and radiographic follow-up was 9.2 years (range 5.0–21.1 years). Every preoperative and postoperative hip radiograph was assessed to determine the degree of osteoarthritis according to the Tönnis classification. Survivorship was analyzed by multistate modeling, enabling assessment of progression through the Tönnis grades rather than just individual transitions as with Kaplan–Meier techniques.

At the time of final follow-up, 144 patients (50%) had progressed at least 1 Tönnis grade, with 42 patients (14.6%) undergoing total hip arthroplasty. The mean number of years spent in each Tönnis grade following PAO was 19 for Tönnis grade 1, 8 for Tönnis grade 2 and 4 for Tönnis grade 3. The probability of progression to total hip arthroplasty increased significantly on the basis of a higher initial Tönnis grade (*P* < 0.001). The most marked difference occurred between Tönnis grade 0 or 1 and Tönnis grade 2; for Tönnis grade 1, the probability of progression to total hip arthroplasty at 5 and 10 years was 2 and 11%, respectively, compared with 23 and 53%, respectively, for Tönnis grade 2.

The authors concluded that PAO effectively alters the natural history of DDH. Precise radiographic progression based on the Tönnis grade can now be used to ascribe prognosis for the native hip. Importantly, this investigation demonstrates a stark increase in progression to total hip arthroplasty within 10 years of PAO for patients with preoperative Tönnis grade 2 osteoarthritis compared with those with Tönnis grade 0 or 1 osteoarthritis.

## STRUCTURED-MENTORSHIP PROGRAM FOR PAO RESULTED IN FEW COMPLICATIONS FOR A LOW-VOLUME PELVIC SURGEON

Considering the complexity of the Bernese PAO with a substantial learning curve, the authors based in Adelaide, Australia proposed to test whether a continuous structured program of distant mentoring offers any benefit [6]. They sought to examine a structured, distant-mentorship program of a low-volume surgeon in a geographically remote setting.

The purposes of this study were (i) to identify the clinical results of PAO performed in a remote-mentorship program, as determined by patient-reported outcome measures and complications of the surgery; (ii) to determine radiographic results, specifically postoperative angular corrections, hip congruity and progression of osteoarthritis; and (iii) to determine worst-case analysis of PAO survivorship, defined as non-conversion to THA, in a regionally isolated cohort of patients with a high rate of follow-up.

Between August 1992 and August 2016, 85 PAOs were undertaken in 72 patients under a structured, distant-mentorship program. The patients were followed for a median of 5 years (range 2–25 years). There were 18 males (21 hips) and 54 females (64 hips). The median age of the patients at the time of surgery was 26 years (range 14–45 years). One patient was lost to follow up (two PAOs) and one patient died as a result of an unrelated event. Patient-reported outcome measures and complications were collected through completion of patient and doctor questionnaires and clinical examination. Radiographic assessment of angular correction, joint congruity and osteoarthritis was undertaken using standard radiology software. PAO survivorship was defined as non-conversion to THA and is presented using worst-case analysis. The loss-to-follow up quotient-number of patients lost to follow up divided by the number of a patients converted to THA-was calculated to determine quality of follow up and reliability of survivorship data.

The median preoperative Harris hip scores of 58 (range 20–96) improved postoperatively to 78 (range 33–100), 86 (range 44–100), 87 (range 55–97) and 80 (range 41–97) at 1, 5, 10 and 14 years, respectively. Sink Grade III complications at 12 months included four relating to the PAO and one relating to the concomitant femoral procedure. The median LCEA correction achieved was 22**°** (range 3**°**–50**°**) and the median correction of acetabular index was 19**°** (range 3**°**–37**°**). Osteoarthritis progressed from a preoperative mean Tönnis grade of 0.6 (median 1; range 0–2) to a postoperative mean of 0.9 (median 1; range 0–3). Six hips underwent conversion to THA: five for progression of osteoarthritis and one for impingement. At 12-year follow up, survivorship of PAO was 94% [95% confidence interval (CI) 85–98%] and survivorship with worst-case analysis was 90% (95% CI 79–96%). The loss-to-follow up quotient for this study was low, calculated to be 0.3.

When PAO is performed using a structured process of mentoring under the guidance of an expert, one low-volume surgeon in a geographically isolated region achieved good patient-reported outcomes, a low incidence of complications at 12 months, satisfactory radiographic outcomes and high survivorship. The authors concluded that a structured distant-mentorship program may be a suitable method for initially learning and continuing to perform low-volume complex surgery in a geographically isolated region.
